# Comprehensive and equitable approaches to the management of neurological conditions in low-and middle-income countries-A call to action^☆^

**DOI:** 10.1016/j.bas.2022.101701

**Published:** 2022-12-02

**Authors:** Camilla G. Aukrust, Roisin McNicholas, Andrea Sylvia Winkler, Walter Johnson, Jogi Pattisapu, Colette White, Vigneshwar R. Veerappan, Ahmed Negida, Kee B. Park

**Affiliations:** a Department of Neurosurgery, Oslo University Hospital, Oslo, Norway; b Department of Community Medicine and Global Health, University of Oslo, Oslo, Norway; c School of Medicine, University of Leeds, Leeds, UK; d Center for Global Health, Department of Neurology, School of Medicine, Technical University of Munich, Munich, Germany; e Center for Global Health, Institute of Health and Society, University of Oslo, Oslo, Norway; f School of Public Health Loma Linda University, Loma Linda, CA, USA; g College of Medicine, University of Central Florida, Orlando, Fl, USA; h Tulane University School of Medicine, Tulane University, New Orleans, LA, USA; i Hull York Medical School, University of York, York, UK; j Global Neurosurgery Initiative, Program in Global Surgery and Social Change, Harvard Medical School, MA, USA; k Faculty of Medicine, Zagazig University, Egypt; l Program in Global Surgery and Social Change, Harvard Medical School, MA, USA

**Keywords:** Neurology, Neurosurgery, Pediatric, Health systems, Policy recommendations

The recent launch of the *Intersectoral Global Action Plan on Epilepsy and other Neurological Disorders 2022–2031* (World Health Organization, 2022a) (IGAP) involves a 10-year commitment to invest in neurological disorders from the World Health Organization (WHO) and its Member States. Since neurological disorders contribute significantly to morbidity and mortality worldwide, this historic milestone deserves attention from Ministries of Health, policymakers, and other key stakeholders (including patients, their caregivers and civil society at large) (Feigin et al., 2021). Priorities should be directed towards low-income and-middle income countries (LMICs) who carry the highest burden of epilepsy and other neurological disorders (Knauss et al., 2019), while at the same time experiencing a severe lack of specialized neurological workforce and healthcare infrastructure (World Health Organization, 2022b). Almost 80% of people with epilepsy live in LMICs, where the treatment gap ranges from 50 to 75% (World Health Organization, 2022b). Similarly, over the past 4 decades, stroke incidence in LMICs has more than doubled; currently, 80% of stroke burden is in LMICs, who have less than 20% of global resources for prevention and treatment (Owolabi et al., 2018).

Some neurological disorders, such as spina bifida and hydrocephalus, require surgical treatment to minimize disabilities or avoid mortality. Like epilepsy, the incidence of spina bifida and hydrocephalus is unequally shouldered by LMICs, where these conditions place an enormous burden on vulnerable populations and health systems (Dewan et al., 2019). The extensive challenges presented by neurological/surgical disorders demand that policymakers and Ministries of Health base their decisions on sustainable and evidence-based recommendations. This calls on the need for a concerted effort from the broader global health community, beyond the clinical and surgical subdivisions of medical disciplines. It is against this backdrop that the major consensus, originating from Harvard Medical School, Program in Global Surgery and Social Change; *Comprehensive Policy Recommendations for the Management of Spina Bifida and Hydrocephalus in Low-and Middle-Income Countries* (CHYSPR, 2021) (CHYSPR) was published, in November 2021. The advisory group supporting and creating CHYSPR included clinicians and scholars from different disciplines and professions (such as pediatricians, neurologists, neurosurgeons, anesthesiologists, nurses, nutritional and public health experts) originating from 18 different countries, including many LMICs, as well as different professional societies [such as the International Society for Pediatric Neurosurgery (ISPN), the World Federation of Neurosurgical Societies (WFNS), and the Global Alliance for prevention of Spina Bifida F-(GAPSBi-F)].

While IGAP applies epilepsy as an entry point for other neurological disorders, CHYSPR focuses on spina bifida and hydrocephalus as an entry point for pediatric neurosurgical conditions (Kanmounye et al., 2022). These two contemporary flagship policy documents represent coordinated efforts that provide comprehensive, feasible, and evidence-based political roadmaps for the prevention, treatment, and rehabilitation of neurological/neurosurgical conditions in general (IGAP), and spina bifida and hydrocephalus, specifically (CHYSPR). While IGAP has 5 main objectives structured around 6 guiding principles (Fig. 1), CHYSPR provides policy recommendations, which are divided into 6 sections, each section applying the WHO health systems and person-centered framework, and is elaborated on using practical and actionable ideas specific to the LMICs context (Fig. 2).

**Fig. 1:**
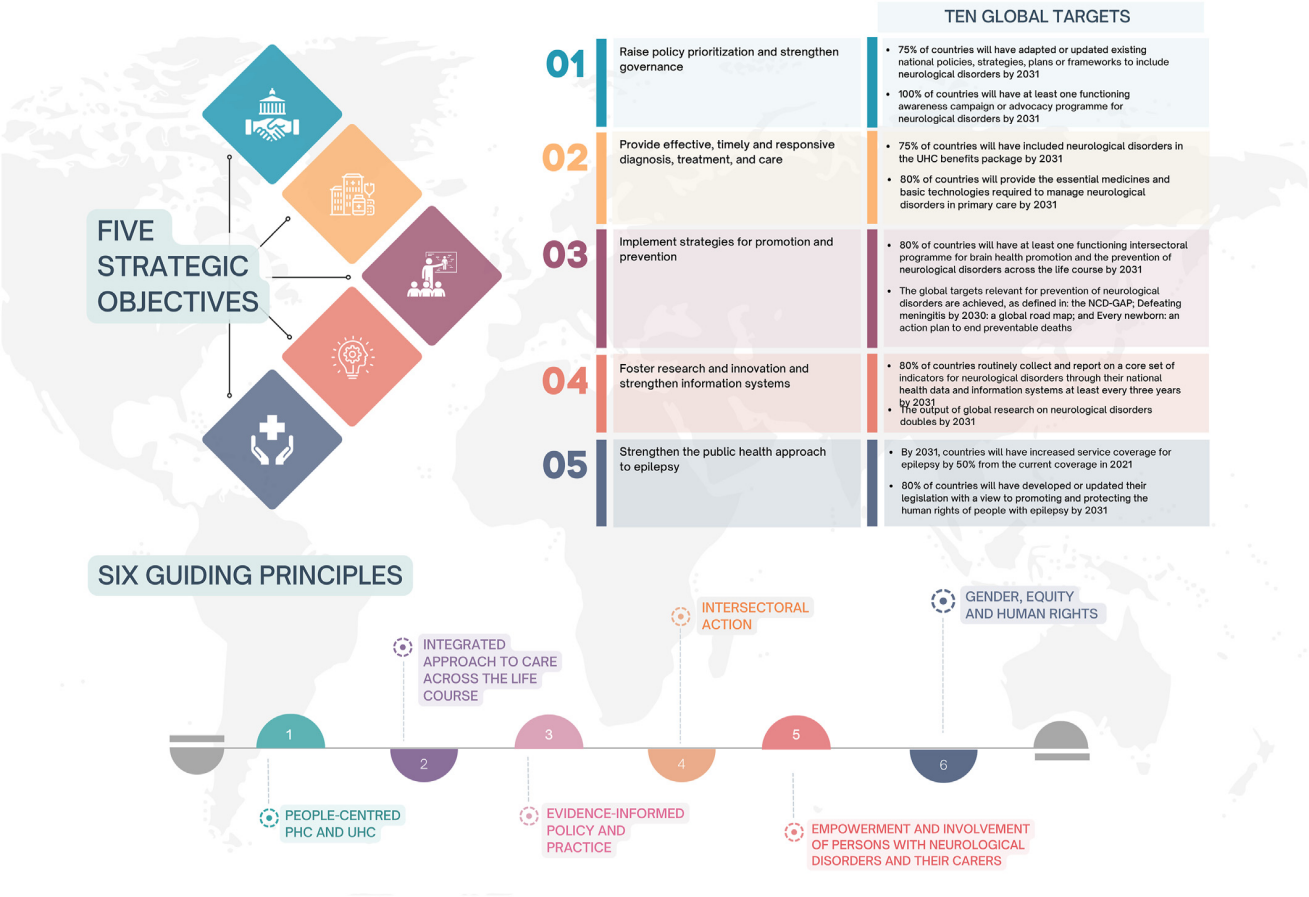
Illustrative presentation of the World Health Organization, Intersectoral Global Action Plan on Epilepsy and other Neurological Disorders, 2022–2031.

**Fig. 2:**
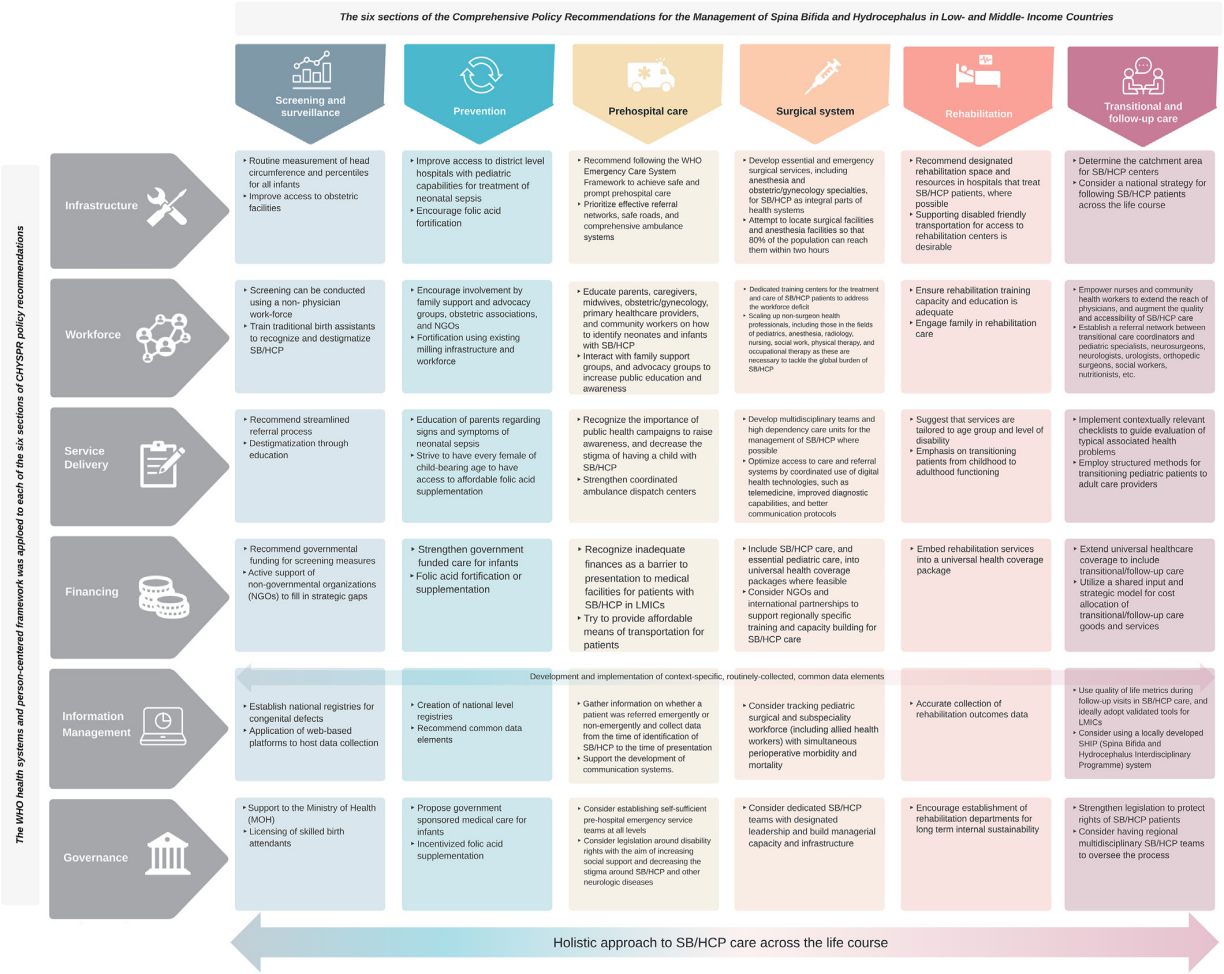
Matrix highlighting selected policy recommendations from the document Comprehensive Policy Recommendations for the Management of Spina Bifida and Hydrocephalus in Low-and Middle-Income Countries.

The documents have numerous parallels and are complimentary in their emphasis of a comprehensive and holistic life-course approach, the importance of interdisciplinary care teams, the need for preventive measures, and challenges related to social stigma and discrimination.

While the artificial separation between neurological and neurosurgical conditions is clinically necessary, it is beneficial to address neurology and neurosurgery collectively, as well as comprehensively, when considering health system strengthening at a policy level. Both disciplines rely on the same strong horizontal health systems and several conditions may be managed medically and/or surgically. For example, resective surgery for drug-resistant epilepsy is efficient and safe (Jetté et al., 2016), and stroke management relies on a surgical facility, whether it is an ischemic stroke in need of thrombectomy or an intracerebral/subarachnoid hemorrhage requiring evacuation of hematoma and/or securing an aneurysm or another vascular anomaly.

Similarly, spina bifida and hydrocephalus are often addressed as (neuro) surgical conditions, and closely linked to other medical/nutritional prevention or treatment strategies. CHYSPR specifically recommends mandatory folic acid fortification and affordable folic acid supplementation for all women of childbearing age to prevent spina bifida. This is in line with an urgent call to action, addressed at the 75th World Health Assembly in Geneva in May 2022, and supported by a publication in the *Lancet Global Health* (Kancherla et al., 2022). Moreover, a major causative factor for hydrocephalus in LMICs is post-infectious, and a recent meta-analysis concluded that, in Africa, this constitutes the main etiology in hydrocephalic children (Aukrust et al., 2022). In sub-Saharan Africa alone, it is estimated that 5.29–8.73 million disability-adjusted-life years (DALYs) are lost every year due to neonatal sepsis, which constitutes a massive public health burden that can result in post-infectious hydrocephalus and other neurodevelopmental impairments (Ranjeva et al., 2018). With more knowledge on routes of infection and microbial origin, one may envision that prevention/medical treatment can replace surgical management in the future.

As the creation and launch of IGAP and CHYSPR are behind us, the crucial phase of dissemination and implementation lies ahead. To this end, the newly released position paper from the WHO, *Optimizing Brain Health across the Life Course* (World Health Organization, 2022c) which is a technical complement to IGAP, serves as an excellent resource that may support the implementation of both IGAP and CHYSPR. An important aspect of this position paper is that it proposes a framework addressing five broad groups of brain health determinants; physical health, healthy environments, safety and security, life-long learning and social connection, and access to quality services (World Health Organization, 2022c). *Optimizing Brain Health across the Life Course* is representative of the accelerated push for increased investments in brain health worldwide, and underlines the significance of intersectoral, interdisciplinary, and multi-stakeholder collaboration.

Both neurological and neurosurgical conditions require action at a policy level. IGAP and CHYSPR not only provide a comprehensive guide on how these conditions can be addressed, but also hold potential for synergistic methods to improve healthcare services outside of epilepsy, hydrocephalus, and spina bifida. We hope national governments and Ministries of Health soon adopt, and importantly contextually adapt, these political strategies and recommendations. We are confident such actions will improve the lives of many, with implications far beyond neurology and neurosurgery.

## Funding sources

This research did not receive any specific grant from funding agencies in the public, commercial, or not-for-profit sectors.

## Credit author statement

Camilla G. Aukrust: Drafting the article.

Roisin McNicholas: Creation of figures.

Andrea Sylvia Winkler: Critical revision of the article.

Walter Johnson: Critical revision of the article.

Jogi Pattisapu: Critical revision of the article.

Colette White: Critical revision of the article.

Vigneshwar R. Veerappan: Critical revision of the article.

Ahmed Negida: Critical revision of the article.

Kee B. Park: Conception. Critical revision of the article.

## Declaration of competing interest

The authors declare that they have no known competing financial interests or personal relationships that could have appeared to influence the work reported in this paper.
